# Diagnostic and prognostic applications of machine learning in paediatric traumatic brain injury: a systematic review of single and multimodal approaches

**DOI:** 10.3389/fneur.2026.1838137

**Published:** 2026-07-14

**Authors:** Zolisa Nkabinde, Abdullah Laher, Devon Jarvis

**Affiliations:** 1 Department of Emergency Medicine, Faculty of Health Sciences, University of the Witwatersrand, Johannesburg, South Africa; 2 School of Computer Science & Applied Mathematics, University of the Witwatersrand, Johannesburg, South Africa

**Keywords:** machine learning (ML), multimodal data, outcome prediction, paediatric traumatic brain injury (pTBI), prognostication

## Abstract

**Background:**

Paediatric traumatic brain injury (pTBI) is one of the leading causes of global childhood disability. While traditional tools like the Glasgow Coma Scale (GCS) are clinical staples, they often lack the precision required for individualized care. This systematic review evaluates machine learning (ML) and deep learning (DL) models for both the acute detection of injuries and the long-term prognostication of outcomes in pTBI.

**Methods:**

Following PRISMA guidelines and PROSPERO registration (CRD42024510419), a comprehensive search of five databases through May 2026 was conducted to include recent advancements in transformer architectures and gradient boosting. Inclusion criteria focused on paediatric patients from 0 to 18 years, using single or multimodal data (clinical, imaging, and biomarkers) for TBI identification or outcome prediction. Methodological quality was assessed using QUIPS and PROBAST.

**Results:**

Twenty full-text studies (comprising 68,331 subjects) were analyzed. Models were stratified into acute diagnosis (e.g., skull fracture detection, CT-necessity triage) and outcome prognostication (e.g., mortality, 6-month functional recovery). Algorithms such as XGBoost, Random Forest, and CNNs showed potential to outperform traditional regression in specific scenarios, achieving AUROCs up to 0.98 for mortality. However, gains were marginal in low-risk triage, where ML models did not significantly surpass the “no-information rate.”

**Conclusion:**

ML models show significant potential for enhancing pTBI care through improved risk stratification and automated imaging analysis. Future implementation requires a focus on model interpretability (e.g., SHAP values), addressing class imbalances, and conducting external multicenter validation to ensure regional generalizability.

**Systematic review registration:**

https://www.crd.york.ac.uk/PROSPERO/view/CRD42024510419, identifier CRD42024510419.

## Introduction

Paediatric traumatic brain injury (pTBI) represents a paramount global public health challenge, significantly contributing to childhood disability and mortality (1). Quantifying the global incidence of pTBI remains difficult due to reporting inconsistencies; however, evidence suggests that the annual incidence ranges between 47 and 280 per 100,000 children, a rate significantly higher than that observed in adult populations (1, 2). Outcomes of pTBI exhibit extreme variability even among patients with similar injury patterns, resulting in profound disparities in long-term neurocognitive and physical recovery (3). This lack of predictability hinders the development of standardized management protocols and emphasizes the clinical necessity for personalized prognostic strategies tailored to specific paediatric injury profiles (4).

Traditional diagnostic and prognostic staples, such as the Glasgow Coma Scale (GCS) and the Paediatric Index of Mortality (PIM), often lack the granularity required to provide individualized recovery profiles (1, 5). While the GCS is a useful acute assessment tool, it fails to account for specific injury mechanisms or capture complex neurological dynamics and cognitive complications (5). Furthermore, established computed tomography (CT) classification systems, including the Marshall, Rotterdam, Helsinki, and Stockholm scores, were originally designed for adults and frequently rely on linear additive assumptions (6, 7). These models do not sufficiently accommodate the unique anatomical and physiological characteristics of children, such as varying brain developmental stages, which influence recovery potential and outcomes (7, 8).

The rapid evolution of machine learning (ML) and deep learning (DL) offers a transformative approach to these challenges by identifying patterns within high-dimensional, multimodal datasets (2, 9). ML architectures such as Artificial Neural Networks (ANN), Random Forests, and Support Vector Machines (SVM) excel at discovering non-linear correlations among clinical findings, neuroimaging, and biochemical markers (1, 5, 10). Recent evidence has demonstrated the utility of these models in predicting critical outcomes, including 6-month functional recovery and mortality, often surpassing the predictive power of traditional clinical scoring tools (4, 5, 11). There is a pressing need for validated methods to integrate multimodal data including imaging, laboratory indicators, and clinical assessments into personalized prognostic models (2).

Existing machine learning studies in pTBI address heterogeneous clinical objectives, ranging from acute intracranial injury detection and imaging triage to longer-term functional outcome prognostication (5, 6, 12). Several studies have focused on diagnostic or triage-oriented tasks, including automated identification of intracranial abnormalities, detection of skull fractures, or the prediction of clinically significant CT findings to reduce unnecessary radiation exposure (8, 13–16). In contrast, other investigations have evaluated prognostic endpoints such as mortality, discharge disposition, or functional neurological outcomes using measures including the Glasgow Outcome Scale (1, 3–5, 11, 17). Because these studies target different clinical questions, direct comparison of model performance across the literature requires careful interpretation (14).

Diagnostic models predicting acute imaging findings are methodologically and clinically distinct from prognostic models designed to estimate longitudinal outcomes. Accordingly, this review stratifies studies according to their primary clinical objective to improve interpretability and avoid overgeneralization regarding the comparative performance of machine learning approaches.

Previous reviews, including the systematic review by Lampros et al. (18) and the scoping review by Saldaña-Rimarachin et al. (19), have summarized the application of machine learning and artificial intelligence in paediatric traumatic brain injury, particularly in relation to CT-triage, diagnosis, and prognostic prediction models. However, these reviews primarily focused on aggregate model performance and broad thematic mapping of the literature. Important methodological considerations remain insufficiently explored, including the distinction between acute diagnostic systems and longitudinal prognostic models, overlapping patient cohorts, external validation scarcity, multimodal data integration, explainable artificial intelligence frameworks, and adherence to reporting and risk-of-bias standards such as TRIPOD and PROBAST.

Therefore, this review aims to provide a more structured methodological synthesis of the literature. Attention is given to differences in study design, model architecture including XGBoost, CatBoost, and Convolutional Neural Networks, prediction endpoints, and clinical applicability to provide a structured overview of the current evidence base and identify areas requiring further validation and standardization (2, 6, 7). To date, no systematic review has maintained this level of clear stratification between acute detection and long-term functional recovery while evaluating the role of multimodal data analysis and ML algorithms in the paediatric population.

## Methods

### Search

**Figure 1:**
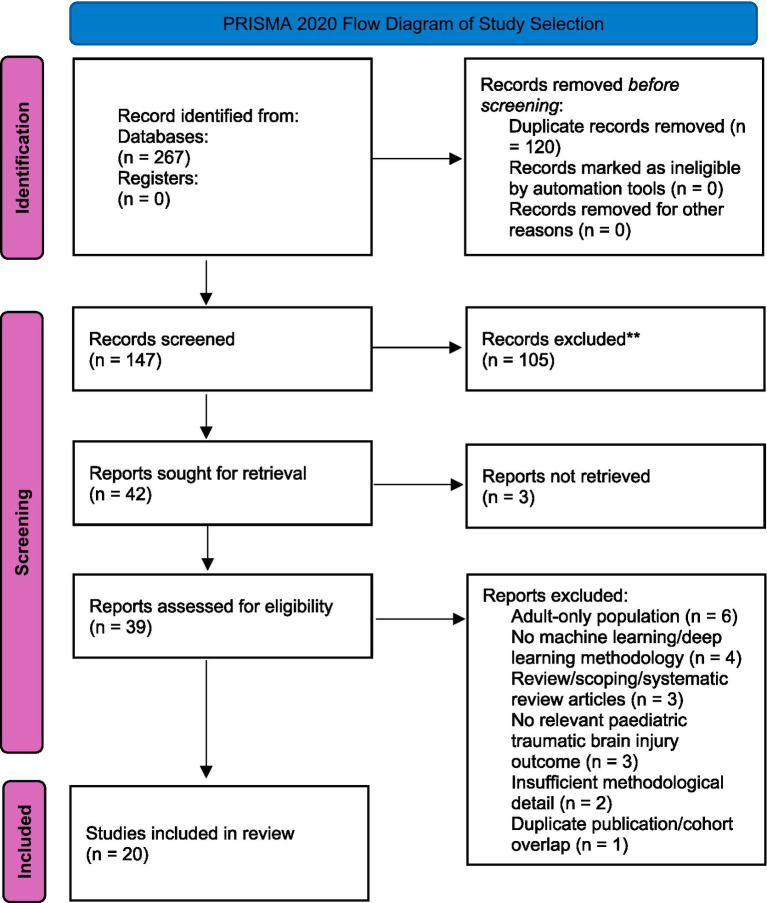
PRISMA flow diagram. Adapted from Page et al. (20), Creative Commons Attribution 4.0 International (CC BY 4.0). Source: PRISMA 2020 flow diagram template downloaded from the PRISMA Statement website.

This review was registered with the Prospective Register of Systematic Reviews (PROSPERO: CRD42024510419) and conducted in accordance with the PRISMA 2020 guidelines (20). A PRISMA flow diagram (Figure 1) was constructed to document study identification, screening, eligibility, and inclusion. The search was conducted across five electronic databases from 1 January 2015 to 31 May 2026: PubMed, Embase, Scopus, Web of Science, and the Cochrane Library. The search period was restricted to 2015 onward to capture contemporary machine learning methodologies relevant to current clinical AI applications. The search strategy incorporated terminology related to specific machine learning algorithms, using the following search terms: (“paediatric traumatic brain injury” OR “pTBI”) AND (“machine learning” OR “deep learning” OR “artificial intelligence” OR “convolutional neural network” OR “CNN” OR “random forest” OR “XGBoost” OR “gradient boosting” OR “support vector machine” OR “SVM” OR “transformer” OR “artificial neural network” OR “neural network” OR “radiomics” OR “classification” OR “predictive model”) AND (“prognosis” OR “prediction” OR “outcomes” OR “diagnosis” OR “triage”). No language restrictions were applied during the search process, and reference lists of retrieved articles were manually screened to identify additional relevant literature.

### Eligibility criteria

Studies were eligible for inclusion if they met the following criteria:

- *Population*: Patients aged 0 to 18 years evaluated for TBI. The study by Kim et al. (7), which used an upper age limit of 19 years, was retained because the cohort was predominantly pediatric, with a median age of 6 years and an interquartile range of 1.75 to 13 years. Since the 75th percentile of the population was well below the review’s 18-year threshold, the inclusion of a small number of late-adolescent subjects was deemed unlikely to alter the densitometric injury patterns identified, particularly given the study’s unique focus on automated intracranial densitometry.
- *Algorithm focus*: Use of ML or DL for either acute injury detection or outcome prognostication.
- *Outcome targets*: Identification of acute intracranial abnormalities (e.g., skull fractures, CT-necessity) or prediction of longitudinal outcomes (e.g., mortality, functional status via GOS/KOSCHI).

*Exclusion criteria*: Studies were excluded if they: (i) were review articles or case reports; (ii) lacked sufficient methodological detail to verify the ML architecture (defined as failing to report at least the algorithm name, validation method, and a primary performance metric like AUC); (iii) focused exclusively on adult populations without a distinct paediatric subgroup analysis; or (iv) were conference abstracts lacking full-text availability; such studies were excluded from primary qualitative synthesis and risk-of-bias assessment but were retained for contextual discussion where necessary.

### Operational definitions and categorization

To improve conceptual consistency, included studies were stratified according to their primary clinical categories:

- *Acute injury detection (diagnosis/triage)*: Models identifying contemporaneous intracranial abnormalities, such as the presence of skull fractures or triaging the necessity of acute CT imaging (8, 13–16).
- *Outcome prognostication*: Models predicting future clinical outcomes, such as in-hospital mortality or 6-month functional recovery (1, 4, 5, 10, 11, 17, 21).

Furthermore, “multimodal data” is defined as the integration of features from at least two of the following categories: clinical (vitals/GCS), radiological (imaging features), biochemical (laboratory markers), or socioeconomic (social determinants of health). Studies using only a single data category, such as imaging alone or laboratory values alone, were classified as single-modality approaches.

### Screening and data extraction

Titles and abstracts were screened by two independent reviewers (ZK and AL), followed by a full-text assessment of eligible records. Discrepancies were resolved through consensus or consultation with a third author. To ensure reporting transparency, de-duplication was performed using Mendeley, followed by manual verification to ensure accurate counts for the flow diagram. Data were extracted into a standardized template encompassing: study design, geographic region, sample size, age range, data modalities, specific ML algorithms, validation methods (internal cross-validation, holdout testing, or external validation), and performance metrics (sensitivity, specificity, AUROC, PPV, NPV).

### Methodological quality and risk of bias

Risk of bias was assessed using the Prediction model Risk Of Bias Assessment Tool (PROBAST). PROBAST assesses four domains: Participant Selection, Predictors, Outcome, and Analysis. The Quality In Prognosis Studies (QUIPS) tool was additionally applied to studies evaluating individual prognostic factors rather than complete prediction models. The 20 studies available as full-text articles underwent full assessment; the three abstract-only studies were excluded from formal PROBAST scoring to prevent misleading ratings based on incomplete data (22–24).

## Results

### Search results and study selection

The literature search identified 267 records across five electronic databases. After removal of 120 duplicate records, 147 titles and abstracts were screened, resulting in the exclusion of 105 records. Full texts were sought for 42 reports, of which 39 were retrieved and assessed for eligibility. A Supplementary Table S1 has been added listing all 19 excluded full-text studies with their corresponding rationales for exclusion. Ultimately, 20 investigations were available as full-text articles and underwent detailed qualitative analysis. Three additional investigations, Greenan et al. (22), Singh and Rakhra (23), and Kaliaev et al. (24) were included as contextual literature based on abstracts alone.

### Characteristics of included studies

#### Study design and geography

The evidence base is predominantly composed of retrospective cohort studies. Geographically, the research is concentrated in the United States (*n* = 7) (5, 8, 11, 12, 14, 17, 25), China (*n* = 3) (2, 3, 9), Thailand (*n* = 2) (15, 21), and South Korea (*n* = 2) (7, 16). Additional contributions originated from Singapore (26), the United Kingdom (10), Canada (1), Japan (13), Portugal (4), and Australia (6).

#### Identification of overlapping cohorts

A significant finding of this synthesis is the concentration of evidence within several key databases, necessitating caution when interpreting the cumulative subject count (*n* = 48,382 unique subjects):

- PECARN dataset: Various subsets of the North American network database (*n* = 43,399) were analyzed by Yadav et al. (12), Rowe et al. (14), Ellethy et al. (6), and Zou et al. (8)
- PECARN framework exception: Miyagawa et al. (13) used the PECARN clinical decision rules but analyzed a geographically distinct local cohort from Japan (*n* = 1,100).
- University of Colorado: Fonseca et al. (4) and Steinmetz et al. (11) used the same institutional dataset of 300 pediatric patients.
- Prince of Songkla University: Two investigations by Tunthanathip et al. (15, 21) used related subsets from a Southern Thailand database (*n* = 964).
- Independent Institutional Cohorts: The remaining investigations used independent datasets from Vanderbilt University (5), Zhengzhou University (2), Seoul National University (7, 16), Zhejiang University (9), and others (*n* = 3,719 unique subjects).

### Acute injury detection and diagnostic triage

#### Imaging-based injury identification

Advanced deep learning architectures demonstrated strong diagnostic performance in radiological interpretation. Choi et al. (16) implemented a YOLOv3 object detection model to identify pediatric skull fractures on radiographs, achieving an area under the receiver operating characteristic curve (AUROC) of 0.922 in internal testing and 0.870 in external validation. Kim et al. (7) reached an AUROC of 0.91 for predicting the need for surgery by integrating automated intracranial densitometry with clinical features. Raji et al. (25) differentiated mild TBI (mTBI) from controls with 94% accuracy using edge density imaging.

#### Clinical triage and CT-necessity prediction

Models designed to minimize radiation exposure yielded mixed results. Miyagawa et al. (13) achieved 95% accuracy using decision trees for infants. Zou et al. (8) introduced a PermFIT-DNN framework that predicted TBI status with an AUROC of 0.794. Ellethy et al. (6) reported that a hybrid RF-ANN model identified mTBI on CT with 99.74% accuracy. Conversely, Rowe et al. (14) found that four algorithms (LR, CART, RF, and GBM) failed to outperform the “no-information rate” in low-risk triage. Tunthanathip et al. (15) achieved an AUROC of 0.80 but noted a low sensitivity of 34%.

#### Automated classification of clinical reports

Yadav et al. (12) validated a hybrid NLP-ML system using a Classification and Regression Tree (CART) to automate the classification of 2,121 free-text radiology reports. The system was robust at identifying negative reports (NPV 99.2%) but demonstrated a lower PPV of 43.6%.

### Outcome prognostication

#### Mortality prediction

Prognostic models for mortality consistently used Random Forest and gradient boosting. Daley et al. (1) used six admission variables to predict mortality with an AUROC of 0.90. Morgan et al. (17) used Classification and Survival Random Forests (CRF/SRF) to predict 6-month mortality after decompressive craniectomy, achieving an AUROC of 0.984. Fonseca et al. (4) used XGBoost to reach an AUROC of 0.91, while Steinmetz et al. (11) found that Extra Trees and Logistic Regression achieved the highest precision (0.98).

#### Functional recovery and complications

Hale et al. (5) used an ANN that achieved a mean AUROC of 0.946 for 6-month functional status, outperforming the Rotterdam score (0.74). Diao and Liang (3) found that a simplified Naive Bayes model (AUC 0.930) outperformed traditional CT grading systems. Tunthanathip and Oearsakul (21) reported an SVM model with 0.95 sensitivity but lower specificity (0.60) for functional outcomes. Zhang et al. (9) predicted postoperative infection with an AUROC of 0.944 by fusing radiomics with clinical risk factors.

#### Prognostication using clinical and biomedical data

Chong et al. (26) used an ANN to predict moderate-to-severe TBI, achieving an AUROC of 0.98, surpassing logistic regression (AUC 0.93). Kayhanian et al. (10) used SVM and admission lactate, H+, and glucose to predict favorable outcomes with 80% sensitivity and 99% specificity. Wei et al. (2) developed an extended XGBoost model incorporating a laboratory indicator model (LIM), reaching an AUC of 0.910 in a validation set.

### Methodological trends across studies

The methodological synthesis of the included investigations highlights a transition from traditional statistical models to complex, non-linear architectures, alongside an increased focus on model transparency and feature importance.

#### Methodological quality and risk of bias

Risk of bias for prediction model studies was primarily evaluated using PROBAST (Table 1), while prognostic investigations were additionally assessed using the QUIPS framework (Table 2). Across the prognostic literature, moderate risk most frequently emerged in study participation, confounding, and statistical analysis/reporting domains.

**Table 1:** PROBAST risk-of-bias and applicability assessment.

Study (year)	Domain 1: participants	Domain 2: predictors	Domain 3: outcome	Domain 4: analysis	Overall risk of bias	Overall applicability
Chong et al. (26)	Low	Low	Low	Moderate	Moderate	Low concern
Yadav et al. (12)	Low	Low	Low	Low	Low	Low concern
Hale et al. (5)	Low	Low	Low	Moderate	Moderate	Low concern
Kayhanian et al. (10)	Low	Low	Low	Moderate	Moderate	Low concern
Rowe et al. (14)	Low	Low	Low	Moderate	Moderate	Low concern
Raji et al. (25)	Moderate	Low	Low	Moderate	Moderate	Low concern
Ellethy et al. (6)	Low	Low	Low	Moderate	Moderate	Low concern
Tunthanathip and Oearsakul (21)	Low	Low	Low	Moderate	Moderate	Low concern
Tunthanathip et al. (15)	Low	Low	Low	Moderate	Moderate	Low concern
Kim et al. (7)	Moderate	Low	Low	Moderate	Moderate	Low concern
Choi et al. (16)	Low	Low	Low	Low	Low	Low concern
Daley et al. (1)	Low	Low	Low	Moderate	Moderate	Low concern
Fonseca et al. (4)	Low	Low	Low	Moderate	Moderate	Low concern
Miyagawa et al. (13)	Low	Low	Low	Moderate	Moderate	Low concern
Zou et al. (8)	Low	Low	Low	Moderate	Moderate	Low concern
Morgan et al. (17)	Low	Low	Low	Moderate	Moderate	Low concern
Wei et al. (2)	Low	Low	Low	Low	Low	Low concern
Zhang et al. (9)	Low	Low	Low	Low	Low	Low concern
Steinmetz et al. (11)	Low	Low	Low	Moderate	Moderate	Low concern
Diao and Liang (3)	Low	Low	Low	Moderate	Moderate	Low concern

**Table 2:** Details of the QUIPS (quality in prognosis studies) tool used to evaluate the methodological quality and risk of bias of the included prognostic studies.

Study (year)	Study design	Participation	Attrition	PF measurement	Outcome measurement	Confounding	Analysis and reporting	Overall risk of bias
Chong et al. (26)	Retrospective case–control	Moderate	Low	Low	Low	Moderate	Low	Moderate
Hale et al. (5)	Retrospective cohort	Low	Moderate	Low	Low	Low	Moderate	Moderate
Kayhanian et al. (10)	Retrospective cohort	Low	Low	Low	Low	Moderate	Moderate	Moderate
Tunthanathip and Oearsakul (21)	Retrospective cohort	Low	Low	Low	Low	Moderate	Moderate	Moderate
Kim et al. (7)	Retrospective pilot	Moderate	Low	Low	Low	Moderate	Moderate	Moderate
Daley et al. (1)	Retrospective cohort	Low	Low	Low	Low	Low	Moderate	Moderate
Fonseca et al. (4)	Retrospective cohort	Moderate	Low	Low	Low	Moderate	Moderate	Moderate
Morgan et al. (17)	Retrospective cohort	Low	Low	Low	Low	Moderate	Moderate	Moderate
Wei et al. (2)	Retrospective cohort	Low	Low	Low	Low	Low	Low	Low
Zhang et al. (9)	Retrospective cohort	Low	Low	Low	Low	Low	Low	Low
Steinmetz et al. (11)	Retrospective cohort	Moderate	Low	Low	Low	Moderate	Moderate	Moderate
Diao and Liang (3)	Retrospective cohort	Low	Low	Low	Low	Moderate	Moderate	Moderate

Study participation was assigned moderate risk in several single-center pilot investigations with small cohorts (*n* < 60) and studies derived from specialized intensive care registries, as these populations may not adequately represent the broader pTBI population. Attrition bias was generally low; however, Hale et al. (5) was rated moderate risk because patients lost to follow-up were assumed to have favorable outcomes, potentially introducing outcome misclassification bias. Prognostic factor measurement consistently demonstrated low risk because investigations relied on objective clinical scores, validated laboratory biomarkers, or automated imaging parameters. Outcome measurement also demonstrated low risk owing to the widespread use of standardized endpoints such as GOS, KOSCHI, mortality, or confirmed postoperative infection status.

Moderate confounding risk was identified in investigations that incompletely adjusted for extracranial injuries or pre-existing neurological disease. Statistical analysis and reporting commonly demonstrated moderate risk due to limited external validation, incomplete calibration reporting, and the use of synthetic oversampling techniques such as SMOTE or ROSE in relatively small datasets without extensive sensitivity analyses (Table 3).

**Table 3:** Summary of the studies included in the systematic review.

Author (year)	Study design	Geographic region	Sample size (*N*)	Age range (yrs)	Data modalities	Specific ML algorithm(s)	Validation method	Sensitivity	Specificity	AUROC	PPV	NPV
Chong et al. (26)	Retrospective case–control	Singapore	195	<16	Clinical (history/PE)	ELM (neural network)	Random train-test split	94.9%	97.4%	0.980	90.2%	98.7%
Yadav et al. (12)	Retrospective analysis	USA	2,121	<18	Text (CT reports)	CART (decision tree)	50/50 train-test split	89.7%	91.9%	NS	43.6%	99.2%
Hale et al. (5)	Retrospective cohort	USA	565	<18	Clinical and radiological	ANN	100-fold resampling	NS	NS	0.946	NS	NS
Kayhanian et al. (10)	Retrospective review	UK	94	≤16	Laboratory (14 markers)	SVM	5-fold CV	80.0%	99.0%	0.900	NS	NS
Rowe et al. (14)	Retrospective	USA	43,399	≤18	Clinical	RF, GBM, CART, LR	5-fold CV and holdout	NS	NS	NS	NS	NS
Raji et al. (25)	Prospective case–control	USA	24	10–16	MRI (edge density)	SVM-PCA	LOOCV	79.0%	100.0%	0.940	NS	NS
Ellethy et al. (6)	Retrospective	Australia	15,271	<18	Clinical and CT findings	Deep ANN	5-fold CV	99.2%	99.9%	NS	99.9%	NS
Tunthanathip and Oearsakul (21)	Retrospective cohort	Thailand	828	<15	Clinical and radiological	SVM, RF, ANN, NB	70/30 train-test split	95.0%	60.0%	0.780	99.0%	100.0%
Tunthanathip et al. (15)	Retrospective cohort	Thailand	964	<15	Clinical	RFC	75/25 train-test split	34.0%	95.0%	0.800	73.0%	80.0%
Kim et al. (7)	Retrospective pilot	South Korea	58	≤19	CT densitometry and clinical	CatBoost	LOOCV	83.0%	78.0%	0.910	NS	NS
Choi et al. (16)	Retrospective multi-center	South Korea	508	<20	Radiographs (AP/Lat)	YOLOv3 (DL)	External validation	78.9%	88.2%	0.870	62.5%	94.4%
Daley et al. (1)	Retrospective cohort	Canada	196	≤18	Clinical, radiological and laboratory	RF (Boruta)	70/30 train-test split	NS	NS	0.910	NS	NS
Fonseca et al. (4)	Retrospective	Portugal	300	0–14	Clinical, laboratory and CT	XGBoost, RF, ANN	5-fold CV and holdout	NS	NS	0.910	NS	NS
Miyagawa et al. (13)	Retrospective	Japan	1,100	<2	Clinical	Decision Tree	80/20 train-test split	NS	NS	0.850	95.0%	NS
Zou et al. (8)	Retrospective	USA	1,429	<2	Clinical	PermFIT-DNN	10-fold CV	4.8%	100.0%	0.790	NS	NS
Morgan et al. (17)	Retrospective	USA	40	Pediatric	Clinical, radiological and laboratory	RF (SRF/CRF)	80/20 train-test split	78.0%	71.0%	0.980	NS	NS
Wei et al. (2)	Retrospective	China	532	<18	Clinical and laboratory	XGBoost	Bootstrapping (1,000 iterations)	NS	NS	0.910	NS	NS
Zhang et al. (9)	Retrospective	China	304	≤14	Clinical and radiomics	CNN-Radiomics	Temporal validation	96.0%	85.5%	0.943	68.6%	NS
Steinmetz et al. (11)	Retrospective	USA	300	0–17.8	Clinical	XGBoost, ET, LR	5-fold CV	85.0%	NS	0.970	91.0%	NS
Diao and Liang (3)	Retrospective	China	103	Pediatric	Clinical and CT scores	Naive Bayes	5-fold CV and holdout	100.0%	65.9%	0.930	NS	NS

ANN, artificial neural network; AP/Lat, anteroposterior/lateral; AUROC, area under the receiver operating characteristic curve; CART, classification and regression tree; CNN, convolutional neural network; CRF, classification and regression forest; CT, computed tomography; CV, cross-validation; DL, deep learning; ELM, extreme learning machine; ET, extra trees; GBM, gradient boosting machine; LR, logistic regression; LOOCV, leave-one-out cross-validation; MRI, magnetic resonance imaging; NB, naive bayes; NPV, negative predictive value; NS, not stated; PE, physical examination; PPV, positive predictive value; RF, random forest; RFC, random forest classifier; SRF, survival random forest; SVM, support vector machine.

#### Methodological summary of PROBAST findings

Most investigations demonstrated low risk of bias in participant selection because they used consecutive enrollment from established trauma registries or institutional pediatric databases. Moderate risk was identified in studies employing case–control methodologies or narrowly selected pilot cohorts, which may limit representativeness of the broader pTBI population. For example, Raji et al. (25) used a case–control design, while Kim et al. (7) included subjects up to 19 years of age, extending beyond the primary pediatric threshold used in this review.

Predictor assessment generally demonstrated low risk of bias because the included variables, such as GCS, pupillary reflexes, biochemical markers, and imaging findings, are routinely used in pediatric neurotrauma and were measured before outcome determination. Outcome assessment was similarly judged to be low risk across most investigations because studies relied on validated functional scales, mortality endpoints, operative requirements, or radiologically confirmed injury patterns.

The analysis domain represented the most common source of methodological concern across the evidence base. Most investigations relied exclusively on internal validation methods, including cross-validation or random split-sampling, without robust external validation on geographically independent cohorts. Calibration assessment was inconsistently reported, with many studies focusing primarily on discrimination metrics such as AUROC while omitting calibration plots or formal goodness-of-fit analyses.

Several pilot investigations also involved relatively small datasets, increasing susceptibility to overfitting and optimistic performance estimation. This concern was particularly relevant in studies reporting exceptionally high predictive performance despite limited cohort sizes. Applicability concerns were generally low because the included investigations aligned closely with the review objectives of acute injury detection, prognostic stratification, and functional outcome prediction in pediatric traumatic brain injury populations.

#### Computational architectures and recurring algorithms

The research landscape is characterized by a diverse array of computational architectures tailored to specific clinical objectives. Artificial Neural Networks (ANNs) have been used extensively for their ability to capture complex, non-linear relationships, serving as the foundation for early prognostic work (5, 26) and recent large-scale diagnostic triage investigations (6). There is a notable preference for ensemble-based methods due to their robustness and inherent feature selection capabilities. Random Forest (RF) models were frequently associated with strong predictive performance for predicting mortality and identifying acute injuries (1, 15, 17), while eXtreme Gradient Boosting (XGBoost) and CatBoost have been increasingly adopted for their high discriminative performance in hospital mortality and long-term functional recovery tasks (2, 4, 7). Conversely, Support Vector Machines (SVM) have been selectively applied in studies focusing on high-dimensional “noisy” data, such as admission serum laboratory values or white matter connectome mapping (10, 21, 25).

#### Impactful predictors across modalities

Across disparate clinical endpoints, a consistent set of clinical and physiological features has emerged as the primary drivers of predictive accuracy. In the realm of outcome prognostication, the Glasgow Coma Scale (GCS), specifically its motor component, and pupillary light reflex (notably fixed or abnormal reactions) were nearly universally identified as critical predictors for mortality and functional recovery (1–3, 5, 17). For triage-oriented models, demographic data such as the specific “days of life” proved essential for infant-specific triage (9, 13). Biochemical signals also provided vital prognostic information, with admission levels of glucose, lactate, pH, and procalcitonin frequently ranking among the most informative features in models utilizing laboratory data (1–3, 9, 10). Radiological markers, including midline shift, cistern status, and specific hematoma locations, provided complementary value, particularly when integrated with clinical data in multimodal fusion models (3, 5, 7, 9).

#### Emerging trend toward interpretability

To address the “black-box” nature of complex machine learning models, recent pTBI literature has shifted toward Explainable AI (XAI) to improve interpretability of model output. Recent investigations have implemented SHAP (Shapley Additive exPlanations) values to visualize how specific features, such as low hemoglobin or high-risk scores, push an individual patient’s prediction toward a favorable or unfavorable outcome (2, 3, 7). Additionally, the Permutation Feature Importance Test (PermFIT) framework has been used to statistically validate the significance of individual clinical features while adjusting for complex confounding structures (8). Other researchers have maintained transparency by utilizing hand-replicable logic through Classification and Regression Trees (CART) or simplified Naive Bayes models, thereby improving transparency and interpretability in clinical decision-making (3, 12, 13).

#### Validation patterns and scarcity of external testing

Most included investigations relied on internal validation methods to estimate performance metrics:

- *Iterative resampling*: 5-fold or 10-fold cross-validation was the standard approach used to ensure model stability across data subsets (3, 4, 6, 8, 15).
- *Bootstrapping*: Advanced internal resampling (often 1,000 iterations) was employed in the most recent literature to provide robust performance estimates and confidence intervals (1–3, 9).
- *External validation gap*: Only four investigations reported robust external or temporal validation on independent patient cohorts, which may limit broader clinical translation and generalizability (6, 9, 12, 16).

## Discussion

The current evidence base for machine learning in paediatric traumatic brain injury (pTBI) demonstrates significant potential for enhancing clinical precision, yet it remains characterized by methodological heterogeneity and fragmented clinical objectives. As identified through this synthesis, existing investigations address disparate clinical pathways, ranging from acute injury detection and diagnostic triage to long-term functional outcome prognostication, which represent distinct clinical objectives and should be interpreted separately (5, 6, 12).

While advanced architectures often demonstrate high discriminative power, their superiority over traditional statistical methods is context-dependent and frequently constrained by the limitations of retrospective, single-center data.

### Stratification of clinical objectives

A critical distinction exists between models designed to identify current physiological states and those intended to predict future recovery. Diagnostic and triage-oriented models primarily aim to automate the detection of traumatic findings or minimize unnecessary radiation exposure. For instance, DL models like YOLOv3 have shown clinical validity in assisting inexperienced physicians with skull fracture detection (16). However, the application of ML for low-risk triage yields mixed results; while some models achieve high accuracy for infants (13), others fail to significantly surpass the “no-information rate” or simple rule-based predictors like a normal GCS and the absence of clinical fracture evidence (14). These findings suggest that ML-derived triage systems may not consistently outperform established clinical decision rules in low-risk presentations, particularly when conventional predictors such as GCS and fracture evidence already demonstrate strong discriminative performance (14, 15). In contrast, prognostic models targeting longitudinal endpoints such as mortality and functional status (GOS/KOSCHI) frequently demonstrated higher predictive performance than established adult-derived CT scoring systems. Studies by Hale et al. (5) and Diao and Liang (3) demonstrate that non-parametric architectures like ANNs and Naive Bayes achieved higher AUROC values than the Marshall and Rotterdam CT scores in predicting 6-month functional recovery. These findings highlight the ability of ML to capture the complex, non-linear relationships inherent in the developing paediatric brain, dynamics that traditional linear additive models may inadequately capture (2, 6).

### Methodological limitations

The synthesized evidence base is subject to several structured methodological limitations that impact the generalizability and clinical translation of the findings. A primary constraint is the reliance on retrospective designs; except for the study by Raji et al. (25), all investigations used retrospective cohorts, which inherently increases the risk of selection and information bias. Furthermore, researchers frequently encountered significant class imbalances due to the low incidence rates of mortality or postoperative infection in pediatric populations, often requiring the implementation of synthetic oversampling techniques such as SMOTE or the ROSE algorithm to achieve the balanced distributions necessary for effective model training (3, 4, 9). Finally, the robustness of several foundational investigations was challenged by small sample sizes; pilot studies involving fewer than 60 subjects faced substantial risks of overfitting, a limitation that may mask the true predictive performance of these models when applied to broader, more diverse clinical populations (7, 17, 25). Publication bias may also influence the evidence base, as studies reporting high predictive performance are more likely to undergo publication than studies demonstrating poor model discrimination.

### Explainable AI (XAI) and interpretability in PTBI models

Interpretability is increasingly recognized as an important consideration in pediatric neurotrauma, where high-stakes clinical decisions regarding surgical intervention, intensive care admission, and end-of-life care must be transparent, justifiable, and aligned with parental expectations. The “black-box” nature of advanced computational architectures, particularly deep learning and ANNs, poses significant clinical risks, as clinicians may hesitate to adopt models that provide a final prediction without revealing the underlying rationale. In the time-pressured environments of emergency medicine and neurosurgery, clinicians require models whose predictions can be linked to clinically recognizable physiological patterns.

Recent studies in the pediatric traumatic brain injury literature have incorporated explainability frameworks to improve model transparency and feature attribution. For instance, SHAP has been used to quantify and visualize how individual features, such as low hemoglobin or high laboratory risk scores, push an individual patient’s risk profile toward a poor prognosis. Similarly, the PermFIT framework was introduced by Zou et al. (8) to provide statistical inference for feature importance, allowing researchers to filter out “nuisance” features and focus triage on the most predictive clinical signals.

The literature reflects an ongoing methodological consideration regarding the trade-off between predictive performance and model transparency. While complex architectures like ANNs often report high AUROC values, they are frequently criticized for being difficult to audit. Conversely, inherently interpretable models, such as CART used by Yadav et al. (12), Simplified Naive Bayes models developed by Diao and Liang (3), and hand-replicable decision trees used by Miyagawa et al. (13), offer transparent logic nodes that clinicians can easily evaluate at the bedside. Recent studies using gradient-boosted ensembles like CatBoost and XGBoost attempt to balance predictive performance with model interpretability by delivering promising predictive power while remaining interpretable through integrated feature importance rankings.

Predictors that repeatedly emerge as highly impactful across these explainable models include the motor component of the GCS, pupillary light reflex, admission glucose, lactate levels, and cranial CT findings such as midline shift and cistern integrity. These findings largely align with established neurotrauma predictors used in clinical decision rules like PECARN, reinforcing the consistency between ML-derived predictors and established clinical risk markers. Despite these developments, most current XAI implementations remain *post hoc* explanatory approaches rather than inherently transparent model architectures. In addition, the literature lacks standardized interpretability metrics and formal clinician usability testing to determine whether these explanations improve real-world clinical decision-making.

### Reporting standards, methodological transparency, and risk-of-bias

Standardized reporting is essential in ML-based clinical prediction to ensure reproducibility, allow for accurate comparison across studies, and facilitate the regulatory approval important for future clinical implementation. The TRIPOD statement and its TRIPOD-AI extension provide the minimal necessary checklist for researchers to describe model development and validation transparently. Furthermore, PROBAST is increasingly recognized as the wildly used for evaluating the risk of bias and applicability of clinical models.

Inconsistent adherence to these standards within the pTBI literature often may obscure methodological weaknesses, potentially leading to overestimated model performance. Ahmed and Shaban (27) identified that reporting quality remains incomplete, particularly regarding full model specification and calibration reporting. Key weaknesses identified across the included pTBI studies include a heavy reliance on retrospective single-center datasets, which limits generalizability, and the frequent use of overlapping cohorts from the same registries, such as the PECARN database, which may overrepresent the apparent breadth of independent evidence.

Small sample sizes in pilot studies (often under 60 subjects) and significant class imbalances regarding mortality or functional outcomes represent further challenges. While techniques like SMOTE or the ROSE algorithm are used to correct these imbalances, they may contribute to optimistic performance estimates if not paired with rigorous external validation. Furthermore, reporting gaps are prevalent regarding calibration assessment, the handling of missing data (often limited to listwise deletion), and the clear justification of decision thresholds.

Although some studies, such as those by Choi et al. (16) and Zhang et al. (9), demonstrated higher methodological rigor through multicenter development and temporal validation, the overall evidence base remains constrained by a lack of independent testing on geographically distinct populations. As advocated by Morgan et al. (17), there is an urgent necessity for the field to adopt the TRIPOD guidelines. Adhering to these standards, alongside PROBAST for risk-of-bias assessment, will ensure that future research provides the transparency required for regulatory approval and real-world patient benefit.

Future research must prioritize multicenter prospective validation to confirm model performance across diverse paediatric populations and resource-limited settings. An abstract-only investigation by Kaliaev et al. (24) suggested that incorporating social determinants of health, such as insurance status and household income, may improve triage accuracy.

## Conclusion

Machine learning demonstrates potential as a decision-support framework in paediatric traumatic brain injury (pTBI), particularly for outcome prognostication, where several studies reported improved predictive performance compared with traditional scoring systems. In contrast, its role in acute diagnostic triage remains less clearly established, with some studies demonstrating limited benefit over existing clinical decision rules. Current progress in the field is increasingly focused on multimodal data integration and the development of more interpretable model architectures. However, substantial methodological limitations persist, including retrospective single-center study designs, overlapping cohorts, heterogeneous outcome definitions, and limited external validation. Although multiple investigations reported high discriminative performance, the generalizability and real-world clinical applicability of these models remain uncertain across broader paediatric populations. Future research should prioritize prospective multicenter validation studies, standardized reporting practices such as TRIPOD, and independent external testing to better define the clinical role of machine learning in paediatric neurotrauma care.

## Data Availability

The original contributions presented in the study are included in the article/Supplementary material, further inquiries can be directed to the corresponding author.
