# Supplementary material for: Diagnostic and prognostic applications of machine learning in paediatric traumatic brain injury: a systematic review of single and multimodal approaches
Source: Front Neurol. 2026 Jul 14;17:1838137. doi: 10.3389/fneur.2026.1838137 (PMC13408385; doi:10.3389/fneur.2026.1838137)
Supplement: Supplementary file 1 [file Table_1.docx]

**Supplementary Table S1: Summary of Excluded Full-Text Articles (n = 19)**

| **First Author (Year)** | **Study Title (Abbreviated)** | **Primary Reason for Exclusion** |
| --- | --- | --- |
| Fan (2025) | Development and validation of a nomogram-based risk prediction model for unfavorable outcomes in pediatric traumatic brain injurya | Conventional nomogram-based prediction model; no machine learning methodology employed |
| García-Arellano (2025) | Comparative performance of CT scales for outcome prediction at discharge in pediatric traumatic brain injury | Comparative CT scoring-system study; no machine learning model development |
| Tang (2024) | Post-traumatic hyperoxia after pediatric TBI | Prognostic factor study using multivariable logistic regression; no machine learning methodology |
| Ferrazzano (2024) | MRI and Clinical Variables for Prediction of Outcomes After Pediatric Severe Traumatic Brain Injury | Conventional multivariable prognostic modeling study; no machine learning methodology |
| Hanalioglu (2023) | Cerebrovascular dynamics after pediatric traumatic brain injury | Physiological prognostic factor study using generalized estimating equations; no machine learning methodology |
| Shaklai (2018) | Prognostic factors in childhood-acquired brain injury | Conventional prognostic factor study; no machine learning methodology |
| Figaji (2023) | An update on pediatric traumatic brain injury | Narrative review article |
| Carlotti (2024) | Management of severe traumatic brain injury in pediatric patients: an evidence-based approach | Clinical guideline/recommendation paper |
| Manet (2025) | Neurosurgical management of the acute phase of adult and pediatric traumatic brain injury | Expert consensus guideline |
| Haydel (2024) | Pediatric Head Trauma | Educational resource (StatPearls chapter) |
| Danehower (2025) | Pediatric and Geriatric Considerations | Narrative clinical review |
| Cannella (2019) | Brain interrupted: Early life traumatic brain injury and addiction vulnerability | Narrative review article |
| Marzano (2021) | Traumatic brain injury biomarkers in pediatric patients: a systematic review | Systematic review |
| Omer (2022) | Birth order and pediatric traumatic brain injury | Epidemiological risk-factor study; no machine learning methodology |
| Au (2017) | Paediatric traumatic brain injury: prognostic insights and outlooks | Narrative review article |
| Donnelly (2017) | Autoregulation in paediatric TBI—current evidence and implications for treatment | Narrative review article |
| Fullerton (2024) | Pediatric Traumatic Brain Injury and Microvascular Blood-Brain Barrier Pathology | Neuropathological case series; no machine learning methodology |
| Dennis (2024) | Neuroimaging Correlates of Functional Outcome Following Pediatric TBI | Narrative review/book chapter |
| Gerlach (2023) | Prehospital care of pediatric traumatic brain injury | Narrative review of prehospital management |

CT = Computed Tomography; MRI = Magnetic Resonance Imaging; TBI = Traumatic Brain Injury.
